# Supplementary material for: Modeling glioblastoma heterogeneity as a dynamic network of cell states
Source: Mol Syst Biol. 2021 Sep 16;17(9):e10105. doi: 10.15252/msb.202010105 (PMC8444284; doi:10.15252/msb.202010105)
Supplement: Supplementary file 6 — Source Data for Figure 5 [file MSB-17-e10105-s004.zip › Figure5A_sourcedata/GSEA_3071/nosigdb_stateI.GseaPreranked.1622795145664/IVY_CT.html]

Details for gene set IVY\_CT[GSEA]

|  || Dataset | state13071 |
| Phenotype | NoPhenotypeAvailable |
| Upregulated in class | na\_neg |
| GeneSet | IVY\_CT |
| Enrichment Score (ES) | -0.6558616 |
| Normalized Enrichment Score (NES) | -3.5229864 |
| Nominal p-value | 0.0 |
| FDR q-value | 0.0 |
| FWER p-Value | 0.0 |
Table: GSEA Results Summary

  

Fig 1: Enrichment plot: IVY\_CT      
 Profile of the Running ES Score & Positions of GeneSet Members on the Rank Ordered List

  

| PROBE | GENE SYMBOL | GENE\_TITLE | RANK IN GENE LIST | RANK METRIC SCORE | RUNNING ES | CORE ENRICHMENT || 1 | MATN2 |  |  | 34 | 1.483 | 0.0039 | No |
| 2 | ID3 |  |  | 86 | 1.152 | -0.0003 | No |
| 3 | EGFR |  |  | 175 | 0.864 | -0.0181 | No |
| 4 | VCAN |  |  | 197 | 0.833 | -0.0165 | No |
| 5 | LFNG |  |  | 228 | 0.772 | -0.0181 | No |
| 6 | SDC3 |  |  | 471 | 0.534 | -0.0843 | No |
| 7 | FIBIN |  |  | 630 | 0.441 | -0.1267 | No |
| 8 | RAMP1 |  |  | 1199 | 0.289 | -0.2913 | No |
| 9 | TTK |  |  | 1620 | -0.270 | -0.4125 | No |
| 10 | NPAS3 |  |  | 1708 | -0.281 | -0.4355 | No |
| 11 | KIF2C |  |  | 1735 | -0.284 | -0.4405 | No |
| 12 | EDNRB |  |  | 1754 | -0.287 | -0.4431 | No |
| 13 | CCNB2 |  |  | 1757 | -0.288 | -0.4410 | No |
| 14 | MSI1 |  |  | 1888 | -0.303 | -0.4764 | No |
| 15 | OLIG2 |  |  | 2044 | -0.324 | -0.5190 | No |
| 16 | HIST1H2AG |  |  | 2090 | -0.333 | -0.5292 | No |
| 17 | GATM |  |  | 2113 | -0.337 | -0.5325 | No |
| 18 | SCD5 |  |  | 2299 | -0.373 | -0.5835 | No |
| 19 | BUB1B |  |  | 2335 | -0.381 | -0.5902 | No |
| 20 | H19 |  |  | 2367 | -0.387 | -0.5957 | No |
| 21 | ABAT |  |  | 2383 | -0.390 | -0.5965 | No |
| 22 | UHRF1 |  |  | 2404 | -0.393 | -0.5987 | No |
| 23 | BUB1 |  |  | 2416 | -0.396 | -0.5982 | No |
| 24 | PCDHGC3 |  |  | 2418 | -0.397 | -0.5947 | No |
| 25 | DSEL |  |  | 2432 | -0.398 | -0.5948 | No |
| 26 | AURKA |  |  | 2467 | -0.404 | -0.6011 | No |
| 27 | BLM |  |  | 2469 | -0.404 | -0.5975 | No |
| 28 | PCBP4 |  |  | 2480 | -0.407 | -0.5967 | No |
| 29 | CDC20 |  |  | 2540 | -0.425 | -0.6101 | No |
| 30 | MEX3A |  |  | 2559 | -0.429 | -0.6113 | No |
| 31 | CDCA8 |  |  | 2695 | -0.470 | -0.6467 | No |
| 32 | PRC1 |  |  | 2705 | -0.472 | -0.6449 | No |
| 33 | MELK |  |  | 2709 | -0.474 | -0.6413 | No |
| 34 | NDC80 |  |  | 2723 | -0.478 | -0.6407 | No |
| 35 | KCNIP1 |  |  | 2756 | -0.487 | -0.6455 | No |
| 36 | CENPF |  |  | 2761 | -0.490 | -0.6421 | No |
| 37 | NUF2 |  |  | 2781 | -0.497 | -0.6430 | No |
| 38 | ASF1B |  |  | 2824 | -0.510 | -0.6506 | Yes |
| 39 | CDCA3 |  |  | 2829 | -0.513 | -0.6470 | Yes |
| 40 | ITGB8 |  |  | 2840 | -0.516 | -0.6450 | Yes |
| 41 | TOP2A |  |  | 2842 | -0.517 | -0.6405 | Yes |
| 42 | CKAP2L |  |  | 2857 | -0.523 | -0.6397 | Yes |
| 43 | SPATA6 |  |  | 2882 | -0.531 | -0.6418 | Yes |
| 44 | PTTG1 |  |  | 2895 | -0.540 | -0.6402 | Yes |
| 45 | CDKN2C |  |  | 2903 | -0.544 | -0.6372 | Yes |
| 46 | KIF4A |  |  | 2953 | -0.574 | -0.6462 | Yes |
| 47 | TACC3 |  |  | 2956 | -0.575 | -0.6414 | Yes |
| 48 | TPX2 |  |  | 3006 | -0.606 | -0.6502 | Yes |
| 49 | GAS1 |  |  | 3009 | -0.607 | -0.6451 | Yes |
| 50 | SOX21 |  |  | 3011 | -0.608 | -0.6396 | Yes |
| 51 | METRN |  |  | 3024 | -0.615 | -0.6374 | Yes |
| 52 | DNMT1 |  |  | 3035 | -0.622 | -0.6345 | Yes |
| 53 | NOVA1 |  |  | 3040 | -0.627 | -0.6298 | Yes |
| 54 | OLIG1 |  |  | 3055 | -0.638 | -0.6279 | Yes |
| 55 | FANCI |  |  | 3074 | -0.656 | -0.6271 | Yes |
| 56 | GTSE1 |  |  | 3083 | -0.663 | -0.6232 | Yes |
| 57 | LMNB1 |  |  | 3121 | -0.692 | -0.6276 | Yes |
| 58 | SMC4 |  |  | 3126 | -0.699 | -0.6222 | Yes |
| 59 | CENPK |  |  | 3127 | -0.700 | -0.6156 | Yes |
| 60 | HIST1H2AH |  |  | 3139 | -0.716 | -0.6122 | Yes |
| 61 | KIF15 |  |  | 3142 | -0.723 | -0.6060 | Yes |
| 62 | CDK1 |  |  | 3149 | -0.727 | -0.6009 | Yes |
| 63 | DBI |  |  | 3165 | -0.746 | -0.5983 | Yes |
| 64 | CDCA2 |  |  | 3189 | -0.767 | -0.5979 | Yes |
| 65 | SALL1 |  |  | 3190 | -0.768 | -0.5907 | Yes |
| 66 | TCF12 |  |  | 3191 | -0.769 | -0.5834 | Yes |
| 67 | CDCA7 |  |  | 3203 | -0.785 | -0.5793 | Yes |
| 68 | BIRC5 |  |  | 3208 | -0.793 | -0.5730 | Yes |
| 69 | FBXO5 |  |  | 3212 | -0.797 | -0.5664 | Yes |
| 70 | UBE2C |  |  | 3225 | -0.812 | -0.5624 | Yes |
| 71 | NCAN |  |  | 3228 | -0.816 | -0.5553 | Yes |
| 72 | BCHE |  |  | 3236 | -0.825 | -0.5496 | Yes |
| 73 | NUSAP1 |  |  | 3237 | -0.825 | -0.5418 | Yes |
| 74 | DTL |  |  | 3249 | -0.848 | -0.5371 | Yes |
| 75 | NLGN1 |  |  | 3259 | -0.865 | -0.5316 | Yes |
| 76 | CENPM |  |  | 3269 | -0.881 | -0.5260 | Yes |
| 77 | SOX9 |  |  | 3271 | -0.883 | -0.5180 | Yes |
| 78 | CCNE2 |  |  | 3276 | -0.889 | -0.5109 | Yes |
| 79 | SOX8 |  |  | 3320 | -0.964 | -0.5145 | Yes |
| 80 | WSCD1 |  |  | 3324 | -0.974 | -0.5062 | Yes |
| 81 | MYBL2 |  |  | 3340 | -1.006 | -0.5012 | Yes |
| 82 | EZH2 |  |  | 3342 | -1.015 | -0.4919 | Yes |
| 83 | RAD51AP1 |  |  | 3345 | -1.030 | -0.4829 | Yes |
| 84 | PBK |  |  | 3354 | -1.046 | -0.4754 | Yes |
| 85 | MCM10 |  |  | 3356 | -1.050 | -0.4658 | Yes |
| 86 | LRRN1 |  |  | 3359 | -1.060 | -0.4565 | Yes |
| 87 | ESCO2 |  |  | 3367 | -1.088 | -0.4483 | Yes |
| 88 | DLL3 |  |  | 3368 | -1.090 | -0.4381 | Yes |
| 89 | NCAPG |  |  | 3370 | -1.098 | -0.4280 | Yes |
| 90 | ATP6V0E2 |  |  | 3380 | -1.133 | -0.4201 | Yes |
| 91 | KCNQ2 |  |  | 3383 | -1.142 | -0.4099 | Yes |
| 92 | GINS2 |  |  | 3386 | -1.156 | -0.3997 | Yes |
| 93 | DHFR |  |  | 3387 | -1.159 | -0.3888 | Yes |
| 94 | HMGB2 |  |  | 3390 | -1.184 | -0.3782 | Yes |
| 95 | CLSPN |  |  | 3391 | -1.184 | -0.3671 | Yes |
| 96 | SOX2 |  |  | 3394 | -1.198 | -0.3565 | Yes |
| 97 | DCLK2 |  |  | 3400 | -1.237 | -0.3463 | Yes |
| 98 | FAM111B |  |  | 3402 | -1.238 | -0.3350 | Yes |
| 99 | RRM2 |  |  | 3420 | -1.297 | -0.3278 | Yes |
| 100 | HES6 |  |  | 3431 | -1.366 | -0.3179 | Yes |
| 101 | KLHDC8A |  |  | 3438 | -1.433 | -0.3062 | Yes |
| 102 | COL9A3 |  |  | 3454 | -1.609 | -0.2955 | Yes |
| 103 | FREM2 |  |  | 3458 | -1.686 | -0.2806 | Yes |
| 104 | FAM181B |  |  | 3460 | -1.707 | -0.2649 | Yes |
| 105 | HIST1H1D |  |  | 3466 | -1.743 | -0.2500 | Yes |
| 106 | HIST1H1B |  |  | 3467 | -1.770 | -0.2333 | Yes |
| 107 | SOX11 |  |  | 3469 | -1.784 | -0.2169 | Yes |
| 108 | PTN |  |  | 3476 | -1.843 | -0.2013 | Yes |
| 109 | LRRN3 |  |  | 3477 | -1.856 | -0.1839 | Yes |
| 110 | SCRG1 |  |  | 3480 | -1.876 | -0.1669 | Yes |
| 111 | ASCL1 |  |  | 3484 | -1.937 | -0.1496 | Yes |
| 112 | POU3F2 |  |  | 3487 | -2.092 | -0.1305 | Yes |
| 113 | ID4 |  |  | 3488 | -2.097 | -0.1108 | Yes |
| 114 | CKB |  |  | 3497 | -2.376 | -0.0909 | Yes |
| 115 | C1orf61 |  |  | 3499 | -2.534 | -0.0674 | Yes |
| 116 | IGFBP2 |  |  | 3509 | -3.484 | -0.0373 | Yes |
| 117 | PTPRZ1 |  |  | 3510 | -4.002 | 0.0003 | Yes |
Table: GSEA details [plain text format]

  

Fig 2: IVY\_CT: Random ES distribution      
 Gene set null distribution of ES for **IVY\_CT**
